# Predictive Association of Pre-Operative Defect Areas in the Outer Retinal Layers With Visual Acuity in Macular Hole Surgery

**DOI:** 10.1167/tvst.10.4.7

**Published:** 2021-04-13

**Authors:** Carmen Baumann, Danilo Iannetta, Ziyaad Sultan, Ian A. Pearce, Chris P. Lohmann, Yalin Zheng, Stephen B. Kaye

**Affiliations:** 1 Royal Liverpool University Hospital, Liverpool, UK; 2 Department of Eye and Vision Science, University of Liverpool, Liverpool, UK; 3 Ophthalmology Department, Hospital rechts der Isar, Technical University of Munich (TUM), Munich, Germany

**Keywords:** macular hole, external limiting membrane, ellipsoid zone, area of defect, visual acuity

## Abstract

**Purpose:**

The purpose of this study was to develop methods to model the external limiting membrane (ELM) and ellipsoid zone (EZ) within the elevated cuff surrounding a macular hole (MH) to determine if the predicted size of the defect in these layers after virtual flattening was associated with the actual postoperative defect and best-corrected visual acuity (BCVA).

**Methods:**

Patients were included who had undergone successful MH surgery. The defects in the ELM and EZ after virtual flattening were modeled using in-house software. Main outcomes were postoperative defects in ELM and EZ at 2 months and BCVA at 12 months.

**Results:**

Fifty-eight patients were included. BCVA improved from 0.87 (0.31) logMAR pre-operatively to 0.26 (0.21) at 12 months (*P* < 0.001). For both the ELM and EZ, the predicted virtually flattened pre-operative defects were associated with the actual postoperative defects at 2 months (R^2^ = 0.33, *P* < 0.01 and R^2^ = 0.50, *P* < 0.01, respectively). There was a significant association of BCVA at 12 months (adjusted R^2^ = 0.85) with the pre-operative modeled area of the defect in the ELM (*P* < 0.01) and to a lesser extent with the defect in the EZ (*P* < 0.01) and base of the MH (*P* < 0.01).

**Conclusions:**

Virtually flattening of the pre-operative defect in the ELM provides important predictive information of visual acuity. Incorporation of tools into commercially available optical coherence tomography (OCT) devices to facilitate such measurements would provide the clinician with important prognostic information.

**Translational Relevance:**

We have developed methodology that can potentially be used to predict the postoperative state of the outer retinal layers and the associated visual outcome in patients undergoing surgery for MH.

## Introduction

Patients who develop a macular hole (MH) have been treated by pars-plana vitrectomy (PPV) since 1991.^1^ Internal limiting membrane (ILM) peeling then became a routine component of MH surgery leading to an increase in anatomic hole closure rates of 85% to 100%, but with lower rates for large (>400 µm) MH.^2^^–^^4^ Surgical techniques, such as the inverted ILM flap technique,^5^ have been developed to improve the anatomic closure rates, but functional success rates have remained less predictable.^2^^–^^7^ Since the introduction of optical coherence tomography (OCT) various factors have been evaluated that may influence postoperative visual outcome, such as the minimum linear diameter (MLD), base diameter (BD), or stage of the MH.^8^

The postoperative status of the ellipsoid zone (EZ) was identified as an important hallmark for visual recovery^9^^–^^13^ with the integrity of the external limiting membrane (ELM) playing a critical role in photoreceptor microstructure, alignment, and survival.^14^ The predictive value, therefore, of the pre-operative size (length) of the defect in the ELM and EZ on visual outcome has been investigated but with conflicting results. Some authors have reported an association of pre-operative EZ^15^^,^^16^ or ELM^17^ defect length and visual outcome, whereas others reported no such association.^18^^,^^19^ Most authors, however, measured the length of the defect usually in one selected meridian rather than the area of the defect. In addition, the largest defect may lie in an oblique axis or the extent of defects in other sections may not be apparent.

Although a MH may have a flat edge, most show an elevated cuff of subretinal fluid at their margin.^20^ Kusuhara et al.,^21^ described a macular hole index (MHI) defined as the ratio of the hole height to the base diameter and reported that the postoperative BCVA in those with a MHI ≥0.5 was better than in those with a MHI <0.5, indicating that the cuff height of a MH is positively correlated with visual outcome. Similar findings were presented by Hillenkamp et al.,^22^ who found a cuff of subretinal fluid to be a positive prognostic factor for functional success after retreatment of persistent MH.

In previous studies, the length of the defect has been measured as the linear distance between the visible endings, parallel to the retinal pigment epithelium (RPE). This does not, however, take into account the contour of these layers within the elevated cuff and the change that may occur in the contour of the ELM and EZ after surgery once the subretinal space disappears and the cuff is flattened and remodeled.

The purpose of this study, therefore, was to model the contours of the ELM and EZ within the elevated cuff surrounding an MH in order to determine if the changes in these layers (both length and area) were predictive of the postoperative defects and BCVA at 12 months.

## Methods

Patients who had successful primary surgery for idiopathic MHs at the Hospital rechts der Isar, Technical University of Munich, Germany, and at The Royal Liverpool University Hospital, UK, between 2016 and 2019 were included. Approval was obtained from the ethics committee or the local institutional review board and complied with the Declaration of Helsinki and informed consent was obtained. Surgery consisted of a 25 or 23-gauge PPV, an ILM peel either with the conventional technique (complete removal of the ILM around the hole) or with an inverted ILM-flap (ILM outside the parafoveal area was peeled but an ILM flap anchored on the edge of the hole was inverted and positioned to cover the MH), and a tamponade with either 16% perfluoroethane (C2F6) or 12% perfluoropropane (C3F8) 12%. All patients had a pre-operative radial OCT scan, and a follow-up at 2 months and at 12 months by which stage they were all pseudophakic. Exclusion criteria were recurrent and secondary MH, high myopia (>8.00 diopter [D]), amblyopia, and any identified vision limiting co-pathology.

**Figure 1.**
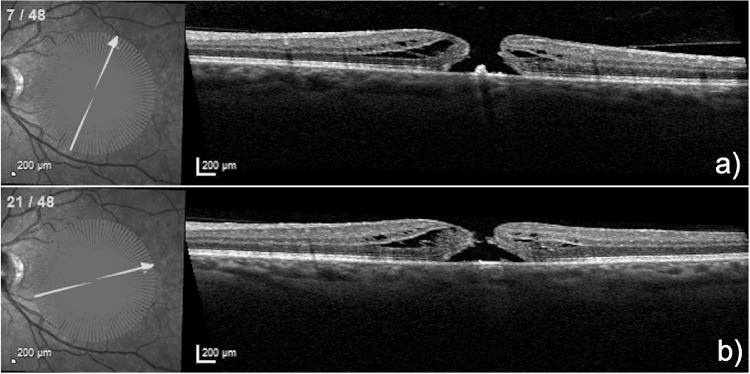
Two different sections of a radial OCT scan in the 1:1 µm mode of a 51-year-old woman who presented with a 12 month history of a MH with an MLD of 363 µm. She underwent combined phaco-vitrectomy with ILM peeling and an inverted ILM flap and gas tamponade. BCVA at 12 months was 0.1 logMAR.

**Figure 2.**
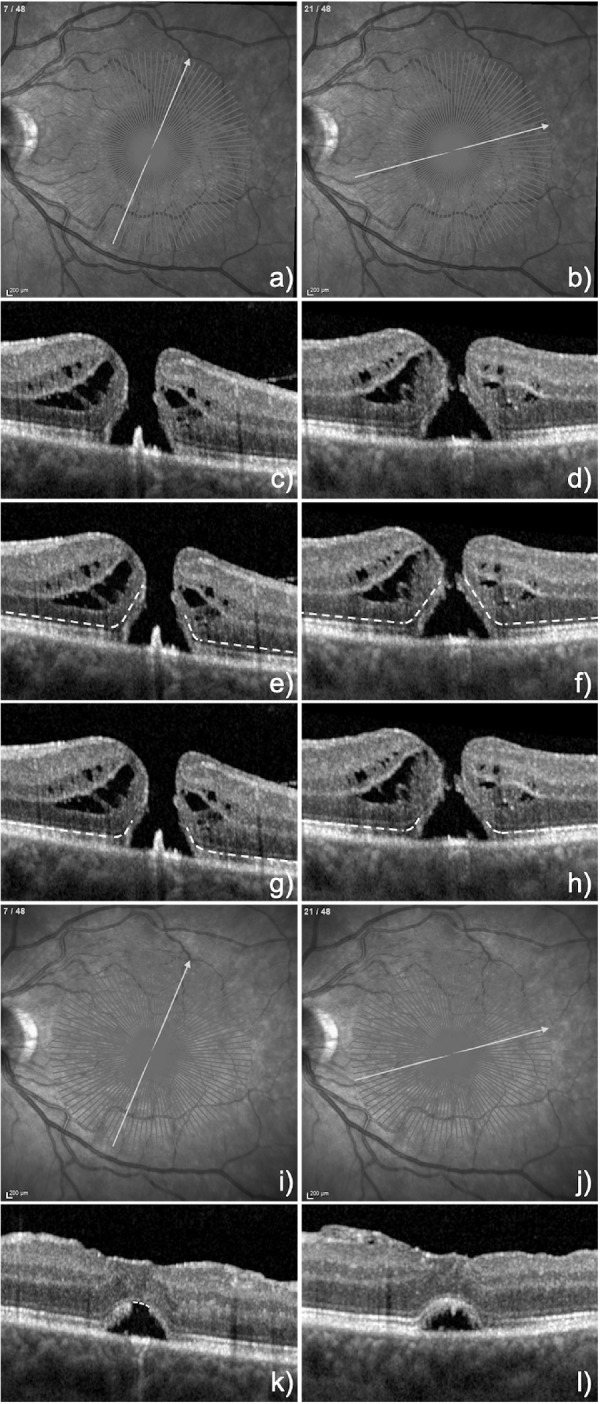
(**A** to **H**) display the same two sections of the radial OCT scan shown in Figure 1 but in the 1:1 pixel mode with **E** and **F** demonstrating the marked ELM layers within the cuffs for each section. Of note is that the ELM in the right cuff in section **E** contains less ELM than in section **F**. **G** and **H** Show the marked residual EZ layers within the cuff, that are approximately equal in extent in both cuffs in both sections. (**I** to **L**) demonstrate the corresponding 2 sections 8 weeks postoperatively, with **K** showing a confluent ELM but a residual defect in the EZ, whereas **L** displays a confluent ELM and EZ.

MHs were assessed using OCT (Heidelberg Spectralis, macular scan with TruTrack active eye tracking) with 24 to 48 radial lines through the center of the MH. All OCT images were converted from the 1:1 µm mode (Fig. 1) into the 1:1 pixel mode (Fig. 2) and uploaded in the graphic program ImageJ (version 2.0.0-rc-69/1.52p) and evaluated by two independent retinal specialists. Postoperative OCT images were taken at 2 months due to the presence of residual gas tamponade within the first few weeks after surgery.

Patient demographics collected included age, sex, duration of symptoms, lens status, pre- and postoperative best-corrected visual acuity (BCVA) logMAR, MH size as MLD and BD. The duration of the MH was based on the duration of symptoms. The main outcomes were the postoperative residual mean defect lengths in the ELM and EZ at 2 months and the BCVA at 12 months.

### Image Analysis

Current spectral-domain (SD) OCT machines conventionally resolve four bands in the outer retina, of which the innermost band is the ELM, and the second band is the EZ. For better visualization, all images were exported and marked in the 1:1 pixel mode and later corrected by using the dimensions of the pixels provided by the Spectralis OCT device (11.76 µm/pixel and 3.87 µm/pixel in the horizontal and vertical directions, respectively). The ELM and EZ bands were identified in the pre-operative macular scans and marked with segmented lines. The marking of the ELM and EZ commenced outside the macular region in a centripetal direction up to the visible endings often within the cuff, irrespective of any attenuation in reflectivity particularly with regard to the EZ band (Figs. 2C–2H). MLD and BD were marked in each scan (not shown). In the postoperative OCT scans at 2 months, the residual defects in the ELM and EZ were marked with segmented lines (Figs. 2K–2L).

All OCT images of 20 patients were marked twice by each rater at least 4 weeks apart to test for intra- and interobserver agreement.

**Figure 3.**
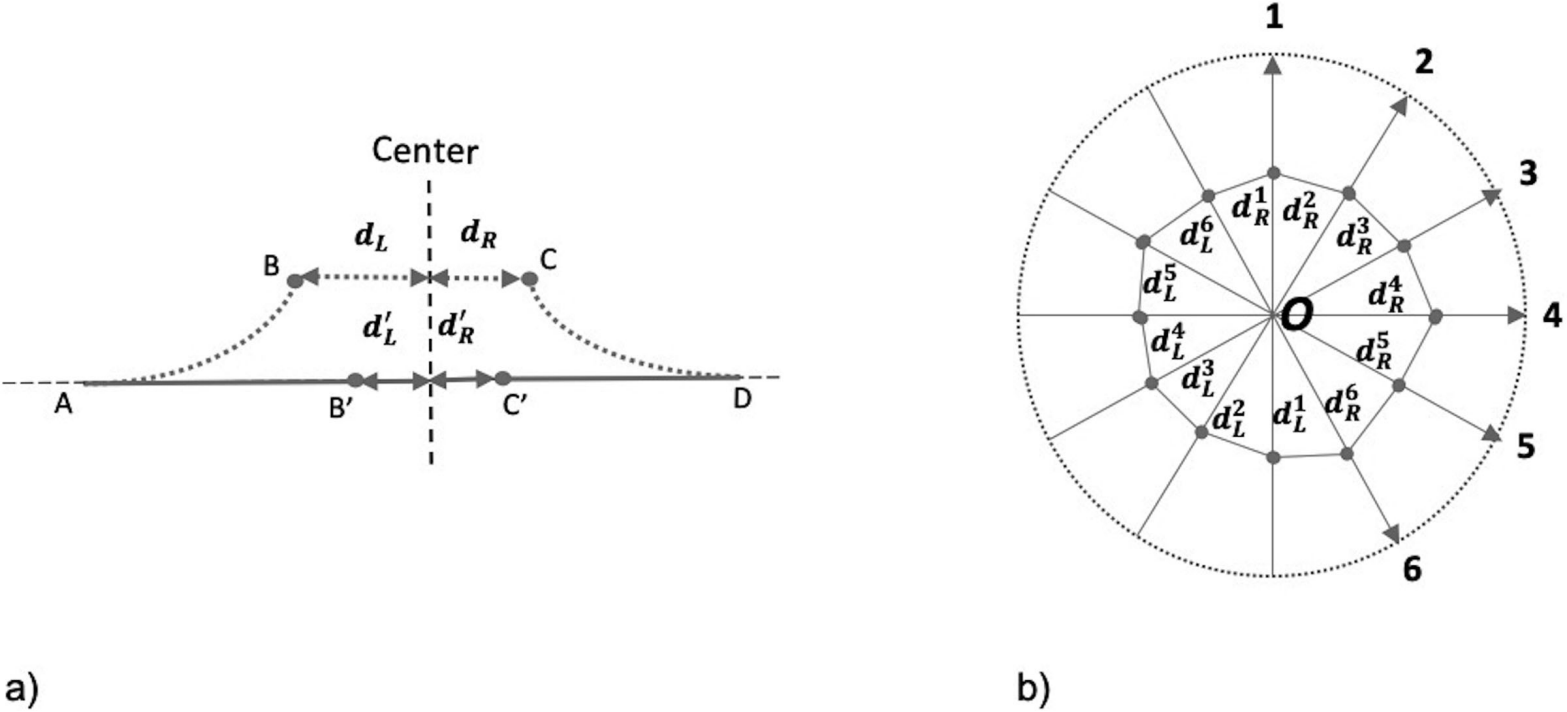
(**A**) Illustration of flattening process in a B-scan. Assume AB and CD (*dotted lines*) is the left and right side, respectively, of a measured feature (ELM and EZ) before flattening. The *d_L_* and *d_R_* are the distances between the end points B and C to the scan center, respectively. AB’ and C'D are after flattening from AB and CD by mapping the arc length of AB to the imaginary reference line of AD. The $d_{L}^{'}$ and $d_{R}^{'}$ are the unflattened distance between the end points B’ and C’ to the scan center. In general, after flattening, the gaps between B’ and C’ would be smaller than that between B and C. (**B**) Illustration of area estimation. Assume a radial scan volume has six B scans for simplicity. For each B scan *i*, we can estimate the gaps on the left and right to the scan center (middle of B scan), $d_{L}^{i}\;$ and$\;d_{R}^{i}$ as described above. An estimate of the enclosed area is made after converting the locations from polar to Cartesian coordinates.

For each B scan, the annotated ELM and EZ layers were analyzed with an in-house program developed by using MatLab (R2019b; MathWorks, Natick, MA). More specifically, for each B scan, the starting (leftmost) and end (rightmost) points of the annotated ELM were used to make an imaginary straight line, which was used as the reference line to virtually flatten the annotated intact ELM. For the left side of the ELM annotation, the shortest distance between its right end and the center of the scan was computed as the “left unflattened radius.” The arc path of the annotated ELM was then flattened along the reference and the shortest distance (“left virtually flattened radius”) was calculated between the right end after virtual flattening and the center of the scan (Fig. 3A). For the right side of the ELM annotation, a similar procedure was applied to obtain “right unflattened radius” and “right flattened radius.” The orientation of the B scan (e.g. θ) and the above radii were used to form orientation/distance pairs for polar coordinates. That is, (θ, left unflattened radius) and (θ +180, right unflattened radius) for the unflattened defect and, similarly (θ, left flattened radius) and (θ +180, right flattened radius) for the virtual flattened defects. These orientation-distance pairs, were then converted into Cartesian coordinates for which the area of the ELM gap was computed (Fig. 3B). The same procedure was applied to the EZ analysis. We then used the horizontal section of the radial OCTs to obtain the left and right radii of the virtually flattened and unflattened defects in the ELM and EZ and computed their lengths by summing the respective corresponding left and right radii of each. To control for differences in MH size, we calculated the pre-operative mean ratio of the ELM and EZ defect area after virtual flattening to the ELM and EZ defect area before virtual flattening.

For the comparative analysis of the defect sizes in the ELM and EZ pre-operatively with 2 months post-operatively, 48 patients with pre- and postoperative radial OCT scans were available and all scans were aligned to compare the corresponding sections pre- and postoperatively. The residual defects in the ELM and EZ were marked with segmented lines and then calculated as mean defect lengths. Because there were many sections that showed already full reconstitution of the ELM and to a lesser extent of the EZ, it would have been incorrect to apply the same method as pre-operatively to connect the defects and calculate the defect area. We have therefore only marked and measured the defect lengths in the ELM and EZ postoperatively in each section and calculated their means.

### Statistical Analysis

Continuous variables were reported as mean ± standard deviation. Those factors and covariates which were significant in a univariate association (*P* < 0.05) were included in a multivariable general linear model as factors and covariates. A Student *t*-test and a Kruskal Wallis test were used with a Bonferroni correction. The statistical package used was SPSS version 25.0 (SPSS, Inc., Chicago, IL).

## Results

Fifty-eight patients were included with a mean age of 67.11 years (7.17 years). Mean duration of symptoms was 5.15 months (3.21 months). Seventeen patients had a conventional ILM peeling and 41 had an ILM flap. No intra-operative complications were reported apart from five patients requiring cryo-retinopexy for retinal breaks. Twelve eyes were pseudophakic at presentation, 14 underwent combined phaco-vitrectomy, and 32 underwent cataract surgery within 10 months after their MH repair. At 12 months, all patients were pseudophakic with an intraocular lens implant in the bag. BCVA improved from 0.87 (±0.31) logMAR pre-operatively to 0.47 (±0.37) at 3 months and 0.26 (±0.21) at 12 months (*P* < 0.001).

The intraclass correlation coefficients (ICCs) for intra-observer agreement for rater 1 and rater 2 were 0.99 and 0.99, respectively. The interobserver agreement between rater 1 and rater 2 was 0.93 and 0.92 for measurement of the EZ and ELM layers.

The mean MLD was 421 µm (±183 µm), BD 844 µm (±297 µm), roof area 0.15 mm^2^ (±0.12 mm^2^), and base area 0.61 mm^2^ (±0.38 mm^2^). The mean pre-operative defect length and area in the ELM was 540 µm (±270 µm) and 0.30 mm^2^ (±0.25 mm^2^). After virtual flattening, this reduced to 480 µm (±280 µm) and 0.26 mm^2^ (±0.25 mm^2^), respectively. The mean pre-operative defect length and area in the EZ was 870 µm (±410 µm) and 0.68 mm^2^ (±0.52 mm^2^), respectively, and 860 µm (±410 µm) and 0.66 mm^2^ (±0.52 mm^2^) after virtual flattening.

The ratio of the area of the defect in the ELM and EZ after virtual flattening to before flattening, was 0.80 (±0.12) for the ELM and 0.96 (±0.04) for the EZ (*P* < 0.01) and was associated with the area of the defect in the ELM (i.e. as the area of the defect increased, so did the ratio [R^2^ = 0.35, β = 0.28, *P* < 0.01) indicating that larger MHs had less elevation of the ELM in relation to the size of the MH.

For both the ELM and EZ, the virtually flattened pre-operative defect lengths were associated with the postoperative defect lengths at 2 months (R^2^ = 0.33 and R^2^ = 0.50; *P* < 0.01 and *P* < 0.01, respectively).

There were no significant associations among BCVA and sex (*P* = 0.40), duration (*P* = 0.18), or the intra-operative use of an ILM flap (*P* = 0.49). The BCVA at 12 months was significantly associated with pre-operative BCVA (*P* < 0.01), age (*P* = 0.01), and the pre-operative lengths of the defects in the ELM (R^2^ = 0.63, *P* < 0.01) and EZ (R^2^ = 0.48, *P* < 0.01), MLD (roof length; R^2^ = 0.48, *P* = < 0.01), and base diameter (R^2^ = 0.45, *P* < 0.01).

**Figure 4.**
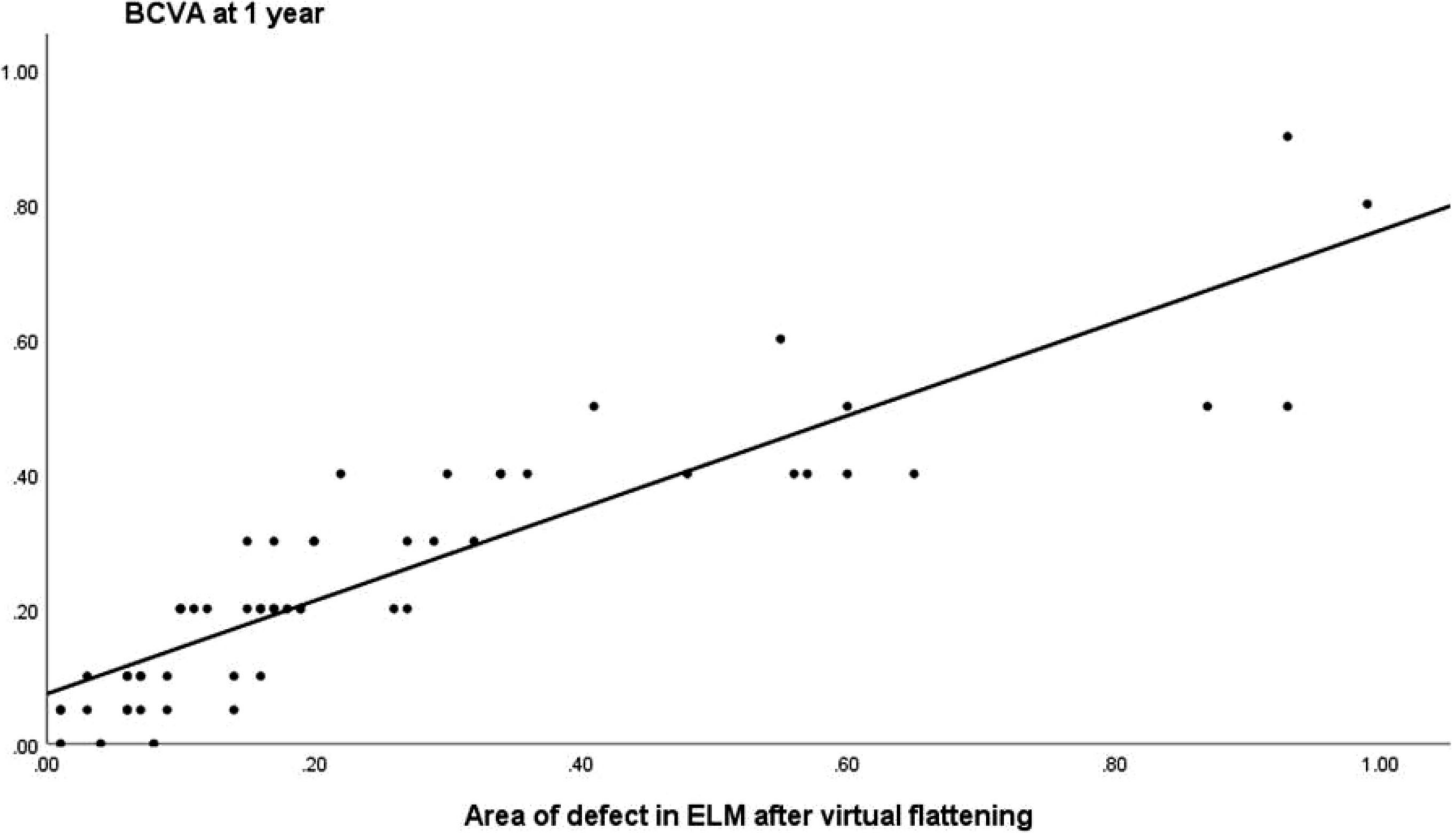
The correlation of BCVA at 12 months with the pre-operative defect areas in the ELM after virtual flattening was 80% (R^2^ = 0.80, *P* < 0.01; *n* = 58).

This predictive association with the BCVA at 12 months, however, was much higher for the pre-operative areas of the defects in the ELM (R^2^ = 0.80, *P* < 0.01; Fig. 4) and EZ (R^2^ = 0.60, *P* < 0.01), roof area (R^2^ = 0.55, *P* < 0.01), and base area (R^2^ = 0.51, *P* < 0.01) than the respective lengths of the defects.

**Figure 5.**
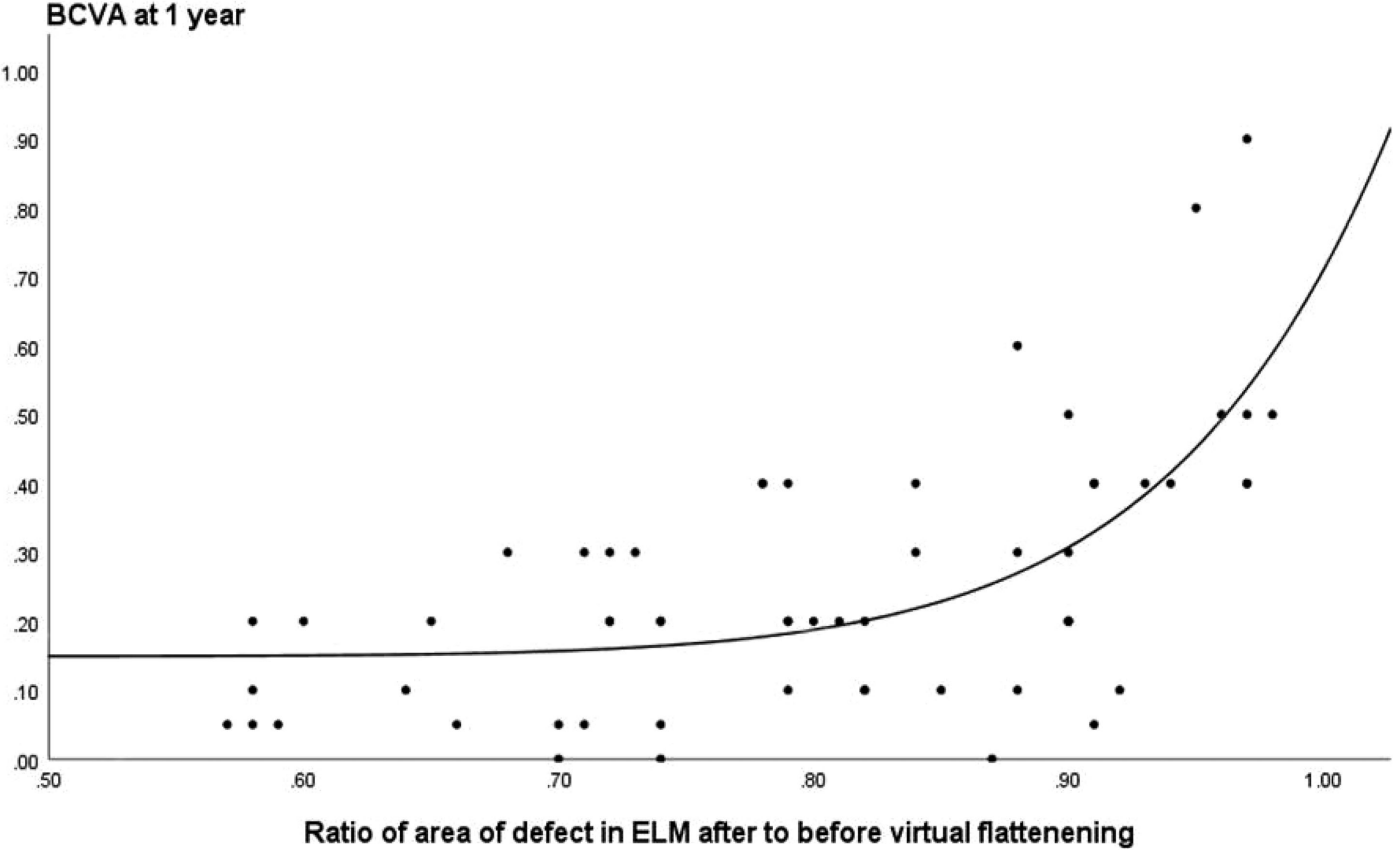
A ratio of the pre-operative defect area in the ELM after virtual flattening to before flattening >0.8 was associated with a significant drop in the BCVA at 1 year (*n* = 58).

A ratio of the ELM defect area after virtual flattening to before flattening > 0.8 was associated with a significant drop in the BCVA at 1 year (Fig. 5).

The ratio of difference in the area of the defect in the ELM (flattened to unflattened) to the area of the defect (i.e. [ELM defect area unflattened – ELM defect area flattened] / [ELM defect area unflattened]) was predictive of the BCVA (R^2^ = 0.42, *P* < 0.01). A higher ratio was associated with a better BCVA outcome at 1 year.

In the general linear model, there was a significant association of the BCVA at 12 months with the pre-operative BCVA (*P* = 0.003), the area of the defect in the base of the MH (*P* = 0.03), and in particular with the area of the defect in the ELM after virtual flattening (*P* < 0.01), with an adjusted R^2^ of 0.85 (*P* < 0.01). The area of the defect in the ELM provided a better predictive association in the model with BCVA than the EZ (adjusted R^2^ = 0.77, *P* < 0.01). There was no significant association of BCVA at 12 months with the MLD (*P* = 0.89), area of the roof defect of the MH (*P* = 0.97), or age of the patient (*P* = 0.98).

## Discussion

Current SD OCT machines conventionally resolve four hyper-reflective bands in the outer retina. The innermost band is the ELM, a linear aggregation of junctional complexes between Muller cells and the photoreceptor inner segments (IS). The ellipsoid region of the photoreceptor IS is densely packed with mitochondria, constituting the second hyper-reflective band on OCT, known as the EZ.^23^ Despite the ELM band typically being thinner and fainter, with current OCT it can be observed more consistently than the EZ, especially underneath and within the cuff surrounding an MH, whereas the EZ band starts to lose some of its reflectivity.^9^^,^^24^^–^^26^ Determination of the defect margins of the EZ involves a greater degree of subjectivity than the ELM.

Itoh et al.^18^ described a “no EZ (IS/OS) band” beyond the edge of the fluid cuff and Oh et al.^9^ defined the boundary of the EZ (IS/OS) defect as the point, where its reflectivity was 2 standard deviations lower than the normalized central mean. Grigoropoulos et al.^16^ defined the EZ (IS/OS) defect as the complete absence of the band, as we have done in this study. Using this definition for marking the EZ, the ICC between two raters was excellent.

The association between pre-operative and postoperative integrity of the ELM and EZ and the postoperative BCVA has been studied but with conflicting results. Chang et al.^19^ did not find a correlation between pre- and postoperative EZ (IS/OS) length defects (*n* = 17). In contrast, we found a reasonable (i.e. a 50%, R^2^ = 0.50, *P* < 0.01) association between the mean pre- and postoperative defect in the EZ at 2 months. The correlation between the mean pre- and postoperative defect in the ELM at 2 months was lower than for the EZ (i.e. 33%, R^2^ = 0.33, *P* < 0.01), which is predominantly attributable to the fact that in most patients the ELM had already been fully restored by 2 months.

Grigoropoulos et al.^16^ selected one of six radial scans in patients with MHs that showed the widest defect in the EZ (IS/OS). They found a negative correlation between the pre-operative EZ defect length and the postoperative EZ integrity at 12 months, the latter being positively correlated with BCVA at 12 months (*n* = 46). Inoue et al.^10^ estimated the postoperative EZ (IS/OS) defect area by multiplying the horizontal and vertical defect lengths at 12 months and found a significant association with postoperative BCVA (*n* = 54). Houly et al.^23^ considered the mean of the defect lengths obtained from horizontal and vertical measurements and showed a linear correlation of the defect in the pre-operative length of 28% (r = 0.53) in the ELM and 11% (r = 0.33) in the EZ with BCVA at 6 months, with the pre-operative length of the ELM defect being the strongest predictor of postoperative BCVA at 6 months (*n* = 52).

We found significant correlations between the pre- and postoperative defects in the ELM and EZ, and the postoperative BCVA at 12 months, which were particularly evident for the defect areas in these layers after virtually flattening. If the attenuated EZ within the cuff had lost its function, we would not have expected the BCVA to be associated with the attenuated margins of the EZ. A lower density of remaining IS or a lower density of mitochondria within the IS may be sufficient to serve functional regeneration after surgery, but may be not able to produce a hyper-reflective distinguishable band on OCT.

The shape and contours of MHs vary significantly within OCT sections. Oh et al.^9^ measured the EZ (IS/OS) defect in horizontal OCT images and the area of the EZ defect in images of the fundus of the eye. They demonstrated that whereas the EZ defect lengths correlated with functional outcome, the irregular shape of the EZ defect area explained why postoperative BCVA correlated more significantly with the measured areas (*n* = 23).

To determine the area of the defect in the EZ and ELM, therefore, we used all sections of the OCT scans. This obviates the need to try and select the most informative meridian of the OCT scan. Although the sections provided by the OCT are not continuous, the method we used lessens the potential for biasing a particular meridian. We found that for all parameters (EZ, ELM, BD, and MLD) the association with postoperative BCVA at 12 months was much greater using the area rather than the length of the defect. For example, in the multivariable model, the association with postoperative BCVA, including the pre-operative area of the defect, was 80% for the ELM and 60% for the EZ, respectively.

To our knowledge, the current study is the first to model the OCT components of the ELM and EZ elevated within the cuff and their contribution to the reduction of the measured defect lengths and areas in the ELM and EZ after virtual flattening. The residual pre-operative defect sizes after virtual flattening were the best predictors of the integrity of the ELM and EZ at 2 months and of BCVA at 12 months. This is in keeping with the association of the integrity of these layers and BCVA shown by other authors.^13^^,^^16^^,^^25^ If virtual flattening reflects the reduction in the size of the gap in the EZ and ELM that would occur with successful surgical hole closure, then it is this residual gap that would still need to be restored. The residual components of the ELM and EZ within the cuff may explain why some studies reported a correlation of final BCVA with cuff height or MHI, depending on how much of these layers were included within the cuff.

The importance of the elevation of the layers within the cuff of an MH was reflected in the ratio of the difference of the flattened to unflattened defect in the ELM to the area of the defect, and the postoperative BCVA. That is the higher the ratio (the smaller the area of the defect and the greater the ELM within the cuff) the better the BCVA outcome at 1 year. If the ratio for the ELM exceeded 0.8, the visual prognosis was significantly reduced. One potential explanation would be that the residual elevated layers within the cuff would no longer be sufficient to compensate for the size of the MH once flattened.

We are aware that our study has several limitations. It is reliant on in-house software that is not present in commercially available OCT devices. The number of patients included is still relatively small and the marking of OCT sections are subjective. In addition, although we have shown a correlation between the virtually flattened defects and actual defect size at 2 months, it is unclear if virtual flattening accurately reflects the reduction in the size of the defect in the immediate postoperative period. It is not clear how much healing occurs in the EZ and ELM in the initial immediate postoperative period, but this is difficult to assess while the gas tamponade is still covering the macula.

Our study has two important translational clinical findings. First, measuring the pre-operative area rather than the length of the defect of the ELM provides important predictive information of the visual acuity outcome. Second, this predictive association can be improved by modeling the virtually flattened area of the defect. Marking multiple sections in the clinical situation is, however, impractical and subject to observer error. We have, however, presented methodology that could be used to develop tools to facilitate the measurement and calculation of the respective defects. The development of such automated tools and incorporation into commercially available OCT devices would present the clinician with information on the respective changes in the retinal layers before and after virtual flattening and improve prognostic assessments. This would be of practical and prognostic importance for the surgeon and patient.
